# Chaos in disease outbreaks among prey

**DOI:** 10.1038/s41598-020-60945-z

**Published:** 2020-03-03

**Authors:** Andreas Eilersen, Mogens H. Jensen, Kim Sneppen

**Affiliations:** University of Copenhagen, Niels Bohr Institute, Blegdamsvej 17, 2100 København Ø, Denmark

**Keywords:** Complexity, Population dynamics, Ecological modelling

## Abstract

Epidemics are highly unpredictable, and so are real-world population dynamics. In this paper, we examine a dynamical model of an ecosystem with one predator and two prey species of which one carries a disease. We find that the system behaves chaotically for a wide range of parameters. Using the allometric mass scaling of animal and disease lifetimes, we predict chaos if (a) the disease is infectious enough to persist, and (b) it affects the larger prey species. This provides another example of chaos in a Lotka-Volterra system and a possible explanation for the apparent randomness of epizootic outbreaks.

## Introduction

Real ecosystems are full of noise and unpredictable dynamics. In systems as unpredictable as the ecosphere, it seems reasonable to look for chaos. Nonetheless true chaos has long been regarded as unlikely in nature. When a model of an ecological system gives rise to chaotic behaviour, it has been taken as an argument against the existence of such a system^1^, although this view has been gradually changing for the past few decades^2^.

A component of ecosystems that is known to frequently be unpredictable is disease^3^. In this paper, we will therefore examine whether the interplay between a generalist predator (here modelled as a predator with two prey species) and a pathogen that affects one of the preys can cause chaotic dynamics. We will prove that under our assumptions it can, and derive the conditions for this to happen, as well as examine on what timescale chaotic effects become noticeable. Our main argument is that chaos may be behind the unpredictability of epizootics.

A truly chaotic system depends so sensitively on initial conditions that it appears to be unpredictable despite being deterministic. Even a tiny change in initial conditions (the proverbial “flap of the butterfly’s wings”) can drastically change the outcome. Dynamics like these are particularly interesting in the context of epidemiology.

One of the most well-known examples of chaos is the logistic map, originally a discrete map model of animal reproduction^4^. Despite some prominent early examples of chaos originating in ecology, the focus in the study of chaos was elsewhere. Fewer instances of chaos were found in ecological models in continuous time such as the one presented here, and the topic therefore received less attention. Nonetheless, chaotic behaviour has been predicted from continuous time mathematical models in some cases^2^. A few important examples include models with two competing prey species^5–8^, *N* competing species^9^, and an omnivore-prey-resource system^10^. See also the review by Hastings *et al*.^2^ for an overview of the earlier work on this topic. A more recent discovery is the fact that chaos occurs even in a simple discrete time Lotka-Volterra system^11^.

More recently, the view of chaos as a solely destabilising factor that leads to ecosystem collapse has therefore mellowed a bit. Earn *et al*.^12^ even suggest that chaos might have a stabilising effect by desynchronising separate ecosystems and thus enabling species re-immigration, the so-called rescue effect^13^. Chaos is thus increasingly thought to be an inherent dynamic in ecosystems^2^. The study presented here lends further support to this view.

**Figure 1:**
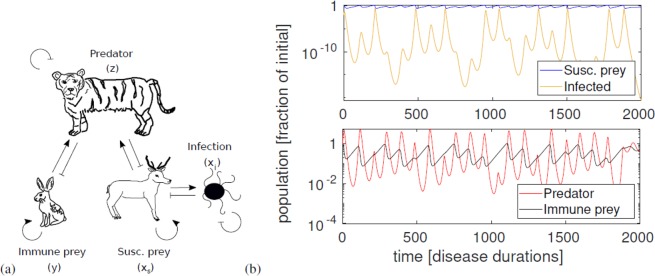
Diagram of the system and time series of the numerical solution. (**a**) A visual representation of the system. Pointy arrows indicate a positive effect, while blunt arrows indicate a negative one. Self arrows indicate reproduction/death that is independent of the other species. Not shown is the interaction between predators and infected individuals, which the predator can eat, although it cannot itself be infected. We expect the number of infected prey to be so small at any given time that it usually does not contribute much to predator growth. The system is still chaotic if we remove the predator reproduction term from eating infected prey. The panel (**b**) shows a simulation of the system. Note especially the large variations in number of infected. Parameters used are *a* = 7*/*400*, b* = 0.0208*, c* = 2*, d* = 0.3098*, R* = 1.5.

Below, we will see that in a system governed by the classical Lotka-Volterra equations, chaos should often be expected when a predator-prey system is exposed to a serious disease. More precisely, we will show that in a system where a generalist predator subsists on two prey species, a disease becoming endemic in one of them can lead to chaos. This system is visualized in Fig. 1, including a typical trajectory for the four variables.

The assumption that the dynamics can be described well by the Lotka-Volterra equations with linear functional responses is fairly restrictive. In nature, it will correspond to an ecosystem where prey equilibrium populations are far from the carrying capacity and it thus makes the applicability of this study more narrow. Nonetheless, some of the basic features of the model presented below are present in nature (e.g. predator-prey oscillations^14^ and periodic epidemics^15^), and we therefore believe it to capture some essential features of real ecosystems, although it may be very approximate.

Most epidemics are notoriously unpredictable^16^, particularly in the cases of zoonoses and vector-borne diseases^17^, and it has previously been considered whether seasonality or noise might drive chaos in the dynamics of certain childhood diseases^16,18,19^. Furthermore, a few papers have examined the potential chaotic behaviour of enzootics in predator-prey ecosystems, usually with infection in the predator^20,21^, but also in the prey^22^. However, to our knowledge, chaos and disease have not been studied in the context of generalist predators, which must be assumed to be more common in nature than entirely specialist predators. Neither has chaos been discovered before in the simplest possible predator-prey-disease models with linear functional and numerical responses and mass-action disease dynamics. It is this discovery that we will present and analyse in this paper. Hopefully, it will further elucidate the role of chaos in ecosystems and the interplay between disease and predation.

## The model

We base our model upon the classical Lotka-Volterra equations^23^, to which we add a second prey species and combine it with the SIR model^24^. To simplify the analysis, we assume that the disease is 100 percent fatal, as recovery with immunity would give rise to a number of new steady states and complex scenarios. This model is in part based on an earlier model^25^, though this had non-linear functional and numerical responses. The older model also exhibits chaotic behaviour, and we will examine it in the supplement.

In order to achieve the most basic system of equations, we assume that all functional and numerical responses are linear, that there is no competition between the prey species, and that there is no carrying capacity on the prey nor the predator. It should however be noted that chaos is still possible in systems with nonlinear functional responses and a prey carrying capacity. We further assume that healthy and infected animals are equally difficult to catch and equally nutritious for the predator. Thus, we arrive at the following equations:

1 $$\begin{array}{rcl}\frac{d{x}_{s}}{dt} & = & {\alpha }_{x}{x}_{s}-\beta {x}_{s}{x}_{i}-{{\epsilon }}_{x}{x}_{s}z\\ \frac{d{x}_{i}}{dt} & = & \beta {x}_{s}{x}_{i}-{{\epsilon }}_{x}{x}_{i}z-\gamma {x}_{i}\\ \frac{dy}{dt} & = & {\alpha }_{y}y-{{\epsilon }}_{y}yz\\ \frac{dz}{dt} & = & {\eta }_{x}({x}_{s}+{x}_{i})z+{\eta }_{y}yz-\delta z,\end{array}$$

where *x*_*s*_ and *x*_*i*_ are healthy and infected populations of the susceptible prey species, *y* is the population of the immune prey species, and *z* is the population of predators. *α*_*x,y*_ are the reproduction rates of the prey species, β is the infection coefficient of the disease, and *γ* is the death rate of infected individuals. Finally, *ε*_*x,y*_ are the coupling constants signifying the rate of individual prey of species *x* and *y* being eaten per predator, while *η*_*x,y*_ are the predator reproduction rates from eating prey of species *x* and *y*, and *δ* is the predator starvation rate in the absence of prey. This is far too many parameters to allow us to get any meaningful information out of the system. Therefore, we rescale it to a timescale and characteristic populations that reflect the dynamics of the system.

First, let $\tilde{t}$ ≡ *γt*, meaning that we will measure time in units of the time it takes an infected prey to die. Thus, the lifetime of the disease is our timescale. We also choose the unit of prey population sizes to be the Lotka-Volterra equilibrium populations in a prey-predator system with only that prey species and the predator. The unit of predator population is similarly chosen as the equilibrium predator population in a Lotka-Volterra system with only the susceptible prey and the predator. Finally, the unit population of infected prey is the same as for susceptible prey. This means that ${\mathop{x}\limits^{ \sim }}_{s}\equiv \frac{{\eta }_{x}}{\delta }{x}_{s},\,{\mathop{x}\limits^{ \sim }}_{i}\equiv \frac{{\eta }_{x}}{\delta }{x}_{i},\mathop{y}\limits^{ \sim }\equiv \frac{{\eta }_{y}}{\delta }y$ and $\tilde{z}\equiv \frac{{{\epsilon }}_{x}}{{\alpha }_{x}}z.$ We then have

2 $$\begin{array}{ccc}\frac{d{\mathop{x}\limits^{ \sim }}_{s}}{dt} & = & a{\mathop{x}\limits^{ \sim }}_{s}-R{\mathop{x}\limits^{ \sim }}_{s}{\mathop{x}\limits^{ \sim }}_{i}-a\,{\mathop{x}\limits^{ \sim }}_{s}\mathop{z}\limits^{ \sim }\\ \frac{d{\mathop{x}\limits^{ \sim }}_{i}}{dt} & = & R{\mathop{x}\limits^{ \sim }}_{s}{\mathop{x}\limits^{ \sim }}_{i}-a{\mathop{x}\limits^{ \sim }}_{i}\mathop{z}\limits^{ \sim }-{\mathop{x}\limits^{ \sim }}_{i}\\ \frac{d\mathop{y}\limits^{ \sim }}{dt} & = & b\mathop{y}\limits^{ \sim }-ac\mathop{y}\limits^{ \sim }\mathop{z}\limits^{ \sim }\\ \frac{d\mathop{z}\limits^{ \sim }}{dt} & = & d({\mathop{x}\limits^{ \sim }}_{s}+{\mathop{x}\limits^{ \sim }}_{i}+\mathop{y}\limits^{ \sim })\,\mathop{z}\limits^{ \sim }-d\mathop{z}\limits^{ \sim },\end{array}$$

where *a* ≡ *α*_*x*_*/γ*, *b* ≡ *α*_*y*_*/γ*, *c* ≡ *ε*_*y*_*/ε*_*x*_, *d* ≡ *δ/γ* and *R* ≡ β x(0)/γ = βδ/γη_x_. *R* (often called *R*_0_ for a given epidemic) is the basic reproduction number of the disease at the initial susceptible prey density *x*_*s*_ = 1. The basic reproduction number is defined as the number of new infections caused by one infected individual dropped into a susceptible population. In real epidemics *R* varies from around 1, the minimum required for the epidemic to start, up to around 18 in extremely infectious diseases such as measles^26^. This rescaling allows us to reduce the number of unknown parameters from nine to five, and also rids us of the coupling constants *η*_*x,y*_ whose size and relationship with *ε*_*x,y*_ are hard to determine. From now on, we will drop the tildes and simply use *x,y,z* etc. to refer to dimensionless variables. With so relatively few parameters, extracting information from the equations should be easy. This is what we will do in the following section.

### Stability analysis

If for some parameter values the system has only one stable fixed point, we will intuitively expect it to not be chaotic. Therefore, we should be able to learn more about the potential for chaos in the system by looking at the stability of the fixed points. The physically possible (i.e. where no populations are strictly negative), nontrivial fixed points of the system 2 are

3 $$(\begin{array}{c}{x}_{s}\\ {x}_{i}\\ y\\ z\end{array})=(\begin{array}{c}1\\ 0\\ 0\\ 1\end{array}),(\begin{array}{c}0\\ 0\\ \frac{b}{ac}\\ 1\end{array}),(\begin{array}{c}\frac{1}{R}\\ \frac{a}{R}\\ 0\\ 0\end{array}),(\begin{array}{c}\frac{b+c}{Rc}\\ \frac{ac-b}{Rc}\\ \frac{b}{ac}\\ \frac{R-1-a}{R}\end{array})$$

For the first fixed point, where the susceptible prey and the predator reach an equilibrium, we have two real and two purely imaginary eigenvalues of opposite sign. The real eigenvalues are (*b* − *ac, R* − 1 − *a*). We expect this fixed point to be (marginally) stable if the real eigenvalues are both negative, i.e. if *b* < *ac* and *R* < 1 + *a*. This translates into a situation where the disease is infectious enough to spread and the immune prey reproduces slowly relative to the susceptible prey.

The second fixed point, where the immune prey and the predator coexist in a Lotka-Volterra equilibrium, similarly has two conjugate imaginary eigenvalues and two real ones, (−1 − *b/c*, *a* − *b/c*). As *b* and *c* are always positive, we expect the first eigenvalue to be strictly negative. The second will be negative and give rise to stability if *b* > *ac*, opposite of the other fixed point.

The third fixed point, an analogue to the Lotka-Volterra steady state but with the pathogen replacing the predator, has two imaginary and two real eigenvalues like the others. One of the real ones is *c*, however, which we know is always positive, so this fixed point is unstable as long as there are two prey species.

**Figure 2:**
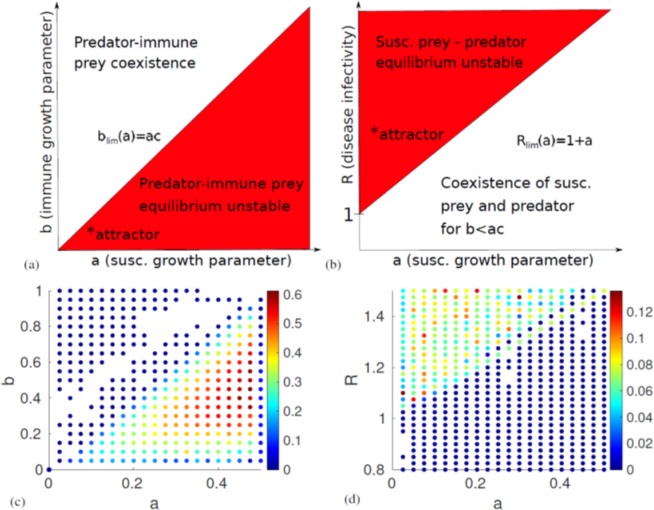
Diagrams of the analytically derived linear stability of the system and the numerical measurements of the Lyapunov exponent. (**a**) Stability of the predator-immune prey equilibrium as a function of the susceptible and immune prey reproduction parameters *a* and *b*. We see that the system will end up in this fixed point if *b* > *ac*. (**b**) Stability of the predator-susceptible prey equilibrium as a function of *a* and the disease basic reproduction number *R*. The system will end up at this fixed point if *b* < *ac* and *R* < 1 + *a*. Thus, we can conclude that chaos is possible only if *b* < *ac* and 1 + *a* < *R*. The approximate location of the chaotic attractor plotted in Fig. 3 is marked with an asterisk. (**c**,**d**) show all parameter values resulting in a Lyapunov exponent *λ* > 0. Note that when looking at the timeseries, we observe no chaos for *λ* < 0.01. In (**c**), the system is expected to become periodic for *a* > 0.5, as 1 + *a* > *R*. This explains the low *λ*-estimate near this value. The colour of the markers indicate the magnitude of *λ*. The diagrams look almost exactly as expected based on linear stability analysis. Parameter values used: **(c**) *c* = 2*, d* = 0.3098*, R* = 1.5, (**d**) *b* = 0.0208*, c* = 2*, d* = 0.3098.

Finally, the fixed point with coexistence of all populations has eigenvalues that are the fourth roots of some function of the parameters. As we know that the fourth roots of any number will be four complex numbers at orthogonal angles in the complex plane, their real parts will always have mixed signs, and this equilibrium will be unstable. Our stability analysis shows that there are two stable fixed points in which the system might end up: If the disease is less infectious than some threshhold *R* < 1 + *a*, and it holds that the immune prey reproduces slower than the threshhold *b* < *ac*, it will end up in the first fixed point, where only the susceptible prey and the predator persist. If the opposite is true, *b* > *ac*, the system will end up in the second fixed point regardless of *R*. Two diagrams of the conditions for stability can be seen in Fig. 2(a,b). On the other hand, if both

*R* > 1 + *a* and *b* < *ac*, none of the fixed points will be stable. We expect that if any chaos occurs, it will happen in this region. However, in order to prove that the system becomes chaotic, we will first have to numerically solve the equations and measure the Lyapunov exponent *λ*.

### Lyapunov exponent and chaotic transitions

In order to determine whether the system is truly chaotic, we use a variant of the Benettin algorithm^27^ to estimate *λ*. Instead of repeatedly measuring and renormalising a unit perturbation vector using the Jacobian, we simultaneously integrate two adjacent trajectories, measure and renormalise their separation vector, integrate again starting with the renormalised separation vector and so on. This method should be equivalent to the Benettin algorithm, as long as we do not change the direction of the separation vector upon normalisation. We thereby determine whether the (small) perturbation vector grows or contracts. In a chaotic system, we should expect two infinitesimally close trajectories to drift apart at an exponential rate, giving a positive Lyapunov exponent. To reduce the number of false positive tests for chaos, we estimate *λ* at two different points in the time series for each set of parameters and choose the lowest of the two values. As can be seen in Fig. 2(c,d) the measurements of the Lyapunov exponent fit with what we expect from linear stability analysis, with a far higher *λ* in regions where chaos is possible. Inspection of the time series of the numerical solutions reveal that the system is in fact not chaotic when the estimated *λ* < 0.01.

**Figure 3:**
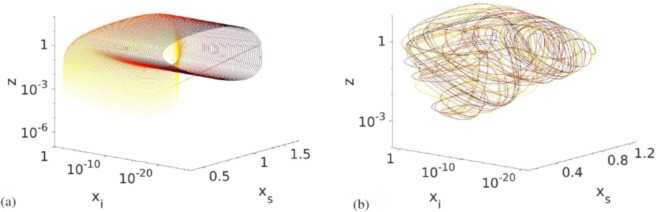
The evolution of the trajectories in *x*_*s*_ − *x*_*i*_ − *z*-space for different values of *R***. (a**) at *R* = 1.025 where the system becomes quasiperiodic near the chaotic trasition. Other parameters were set to *a* = 7*/*400, *b* = 0.0208, *c* = 2, *d* = 0.3098. The fourth dimension, *y*, has been projected out. (**b**) Chaotic attractor for *R* = 1.5. The attractor has a characteristically streaked surface, and we estimate its dimension as *D*_0_ = 3.8 ± 0.1. The colour of the line indicates time, with darker colours signifying later times. Here, we have removed the initial transient.

In Fig. 3 we see (a) the trajectory of the system for *R* close to the transition to chaos where it seems to be quasiperiodic, and (b) a chaotic attractor in *x*_*s*_ − *x*_*i*_ − *z*-space. The attractor has a fairly regular shape with a streaked surface as expected for a structure with a fractal dimension. Here, the variable *y* (immune prey) has been left out for ease of plotting. It was left out because it represents the predator interactions with prey that are less specific than the interactions with susceptible prey. The transition to chaos occurs rapidly and is almost discontinuous. We can therefore rule out the period-doubling route to chaos, as is confirmed by a plot of the peak values of the time series of *x*_*s*_ (Supplemental Fig. 2). Based on the appearance of the trajectory in phase space near the transition (Fig. 3a), we instead believe the transition to happen through a region with quasiperiodic behaviour.

## Discussion

A central question to ask is what parameter values that are realistic. To address this, we will look at the four parameters of the model *a*, *b*, *c* and *d* in terms of the mass of the involved animals. It is a well-known fact that the reproduction rate and metabolism scale with animal mass^28,29^. Disease duration has also been shown to exhibit such scaling^30^. Yodzis & Innes, Dobson, Weitz & Levin, and Eilersen & Sneppen have all attempted to use this to derive information about population dynamics and epidemiology^31–34^. Using the relations found in Peters^28^ and Cable *et al*.^30^, we have that the reproduction rate is $\alpha \approx \frac{1}{400}{m}_{x}^{-1/4}$ [*days*^−1^] and the disease infection period, calculated as the difference between disease death time and time until first symptoms, is *t*_*i*_ = *γ*^*−1*^*≈ km*_*x*_^*1/4*^ [*days*]. Here, *m*_*x*_*, m*_*y*_*, m*_*z*_ are the masses of the susceptible prey, immune prey, and predator (in kilograms), and *k* depends on the disease. For example, *k* is approximately 7 [*days/kg*^1*/*4^] for rabies^30^. We will use this value in our calculations below, as we need an order of magnitude estimate of the coefficient. Note, however, that the disease described here is a hypothetical disease and not in fact rabies.

Similarly, the predator starvation time is approximately its body mass divided by its metabolism, giving *t*_*s*_ = *δ*^−1^≈ $20\frac{{m}_{z}}{{m}_{z}^{3/4}}=20{m}_{z}^{1/4}\,$[*days*]. Now, we can find approximate relations for all the dimensionless parameters:

$$a=\frac{{\alpha }_{x}}{\gamma }\approx \frac{7}{400};\,b=\frac{{\alpha }_{y}}{\gamma }\approx \frac{7}{400}{(\frac{{m}_{x}}{{m}_{y}})}^{\frac{1}{4}};\,c=\frac{{{\epsilon }}_{y}}{{{\epsilon }}_{x}}\approx \frac{{m}_{x}}{{m}_{y}};\,d=\frac{\delta }{\gamma }\approx \frac{7}{20}{(\frac{{m}_{x}}{{m}_{z}})}^{\frac{1}{4}}.$$

To estimate *c*, we have assumed that the number of individuals of a given prey species eaten by the predator will be roughly inversely proportional to the body mass of that species.

We see from these estimates that for a given disease there are in fact only two parameters that matter: The size ratio of the susceptible and immune prey species, and the size ratio of the susceptible prey and the predator. Interestingly, we also see that the condition for chaos *b* < *ac* translates to $\frac{7}{400}{(\frac{{m}_{x}}{{m}_{y}})}^{1/4} < \frac{7}{400}(\frac{{m}_{x}}{{m}_{y}})$, which implies $\frac{{m}_{x}}{{m}_{y}} > 1$. Thus, our model predicts that an epizootic may cause chaos if it affects the larger of a predator’s prey species. As chaos should occur for a wide range of realistic parameter values, it should be fairly common in the wake of epizootics. This of course assumes that our model is a good approximation of nature, which we will discuss below. Furthermore, two-prey one-predator systems should be closer to most real ecosystems than the classical Lotka-Volterra system, since the classical model assumes an entirely specialist predator, which is rare in nature. Even mostly specialist predators are known to have alternative prey in times of scarcity^35^.

What does chaos mean for the ecosystem? The immediate consequence is unpredictable population dynamics. As mentioned in the introduction, the onset of epidemics and epizootics is already known to be unpredictable, but this is often ascribed to stochastic randomness. Our discovery that chaotic dynamics are plausible in ecosystems with three or more species where one is host to a pathogen adds an alternative explanation for this unpredictability.

Based on this model, in the wild one would expect to observe the disease lying dormant for long periods between outbreaks while the infected population is low, and then suddenly and unpredictably breaking out. The model presented here predicts very low minimum infected populations for some parameter values, so in some cases it would probably correspond to real-world pathogens that either die out locally but persist globally, or to pathogens that can survive in some dormant state outside a host. In any case, our assumption that a predator has multiple prey species of different sizes that are susceptible to different diseases should hold almost universally, making our model quite general.

Despite this, some important limitations of the model must be noted. The Lotka-Volterra equations upon which our work is based present a highly simplified and slightly pathological image of ecosystems. One issue is the fact that the classical Lotka-Volterra model with linear functional responses and growth terms gives rise to center equilibria whose orbits depend on initial populations. To avoid this, one could assume that prey species grow logistically. We briefly tried this, and it seemingly makes chaotic dynamics transient by damping oscillations. The conclusion that chaos is widespread is thus dependent on the validity of our assumptions, especially that the Lotka-Volterra model can be used as a rough approximation of real ecosystem dynamics. This is more likely to be true when prey populations are far from the carrying capacity.

In this paper, we have shown that following a disease outbreak, chaos will occur in a two-prey one-predator ecosystem for a wide range of parameters - specifically when the disease affects the larger prey species and is infectious enough to become enzootic. As opposed to previous, similar studies by e.g. Hastings & Powell^5^ or Tanabe & Kumi^10^, chaos occurs in the system studied here even without prey-prey competition or nonlinear functional responses. Our model therefore represents an example of a relatively minimal system that still shows complex dynamics, where the eco-epidemiological models discussed in the introduction require nonlinear responses to show chaotic behaviour. Furthermore, some light has been shed on the interaction between generalist predation and disease. The discussion of how alternative prey species may cause chaos in the context of epizootics has likewise not been explored in the eco-epidemiological studies referenced above. The results presented here are based on the classical Lotka-Volterra model and our conclusions thus depend on its validity, which may be questionable. Nonetheless, the fact that we do not have to impose very tight constraints on parameter values is an argument for the existence and importance of chaos in ecosystems. Most importantly, our results provide one plausible explanation for the apparent unpredictability of epizootics.

## Supplementary information

Supplementary Information.
