# Supplementary material for: Chaos in disease outbreaks among prey
Source: Sci Rep. 2020 Mar 3;10:3907. doi: 10.1038/s41598-020-60945-z (PMC7054530; doi:10.1038/s41598-020-60945-z)
Supplement: Supplementary file 1 — Supplementary Information. [file 41598_2020_60945_MOESM1_ESM.pdf]

Supplementary material to the article "Chaos in disease outbreaks among prey"

Andreas Eilersen[1,*], Mogens H. Jensen[1], and Kim Sneppen[1]

[1]University of Copenhagen, Niels Bohr Institute, Blegdamsvej 17, 2100 København Ø, Denmark
[*]andreaseilersen@nbi.ku.dk

As mentioned in the main paper, Eilersen & Sneppen[1] have previously explored an alternative predator-prey-disease model with non-linear functional and numerical response functions. The proposed system of equations is

$$\begin{aligned}
\frac{dx_s}{dt} &= \alpha x_s(1-(x_s+x_i)/K) - \beta_{xx}x_s x_i - \varepsilon\frac{z}{x_s+x_i+y+k_s}x_s \\
\frac{dx_i}{dt} &= \beta_{xx}x_s x_i - \varepsilon\frac{z}{x_s+x_i+y+k_s}x_i - \gamma x_i \\
\frac{dy}{dt} &= \alpha y(1-y/K) - \varepsilon\frac{z}{x_s+x_i+y+k_s}y \\
\frac{dz}{dt} &= \eta\frac{x_s+x_i+y}{x_s+x_i+y+k_s}z - \delta\frac{k_s}{x_s+x_i+y+k_s}z.
\end{aligned} \tag{1}$$

Here, we have introduced the half-saturation constant k_s and the prey carrying capacity K. It is also worth noting that the constants α, δ, ε, and η now represent maximum values of their respective terms and thus have to be estimated anew. In the cited paper, these quantities are estimated to be

$$\alpha \approx 1/t_g \approx \frac{1}{50}m_x^{-1/4} \quad , \quad \eta \approx \frac{1}{50}m_y^{-1/4} \approx \delta \qquad [1/days] \quad , \quad K \approx 200 m_x^{-3/4} \qquad [prey/km^2],$$

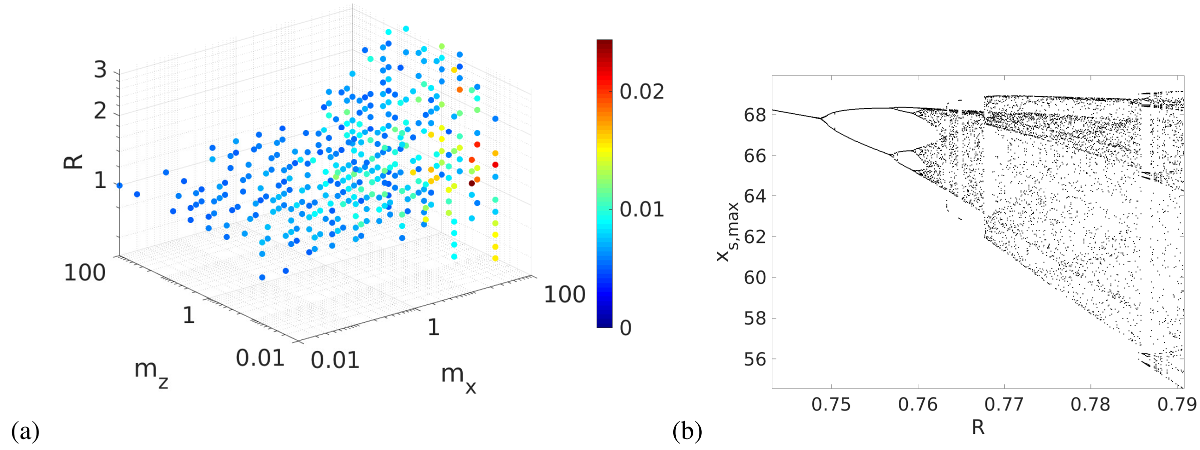

Figure 1. (a) A plot showing all parameter combinations that result in chaos, here defined as $\tilde{\lambda} > 5\cdot 10^{-3}$. The colour of each data point represents the value of $\tilde{\lambda}$. Chaos mainly occurs around $R = 1$ and the range of R that leads to chaos expands at higher prey mass and lower predator mass. In (b), we see a diagram of the peak values of x_s as a function of R, equivalent to a bifurcation diagram. The diagram shows that the chaotic transition happens through period doubling bifurcations. The masses used are $m_x = m_y = 0.4$ kg.

using the allometric scaling method described in the discussion. The model being correct thus depends on the mass parameterisation being correct, but the parameterisation on the other hand allows us to model the system with only three free parameters. We have assumed that both prey species have similar mass for simplicity. Nonetheless, the linear stability analysis of this system is fairly complicated and will not necessarily give us any useful information. We shall therefore restrict ourselves to measuring the Lyapunov exponent of this system as a function of prey mass, predator mass, and disease reproduction number, as well as examining the nature of the transition to chaos.

Even without stability analysis, we expect a transition to take place near $R = 1$, as this is where the epidemic becomes viable at Lotka-Volterra equilibrium populations. We measure the Lyapunov exponent using the same method as above. As the equations are no longer scaled to the natural time scale of the system, we rescale the measured exponents as $\tilde{\lambda} = \lambda/\gamma$. Thereby, we get a new exponent, $\tilde{\lambda}$, which gives the speed of divergence of neighbouring trajectories in units of disease durations. As can be seen in figure 1 (a), chaos in this system exists primarily at low R. The range of R-values where chaos can occur generally grows with prey size and shrinks slightly with predator size.

Figure 1 (b) shows a diagram of the peaks of the susceptible prey (x_s) time series. From this diagram, we see clearly how the transition to chaos takes place through a series of period-doubling bifurcations.

This documents that when using nonlinear functional and numerical responses, chaos can occur in a predator-prey-disease system. It even happens when the prey masses are equal, which we otherwise would not predict using the simpler model with linear response functions. As saturating response functions are probably more realistic in many ecological systems, this result strengthens our argument for the ubiquity of chaos in the wake of disease in predator-prey systems.

Chaotic transition of the system studied in the main paper

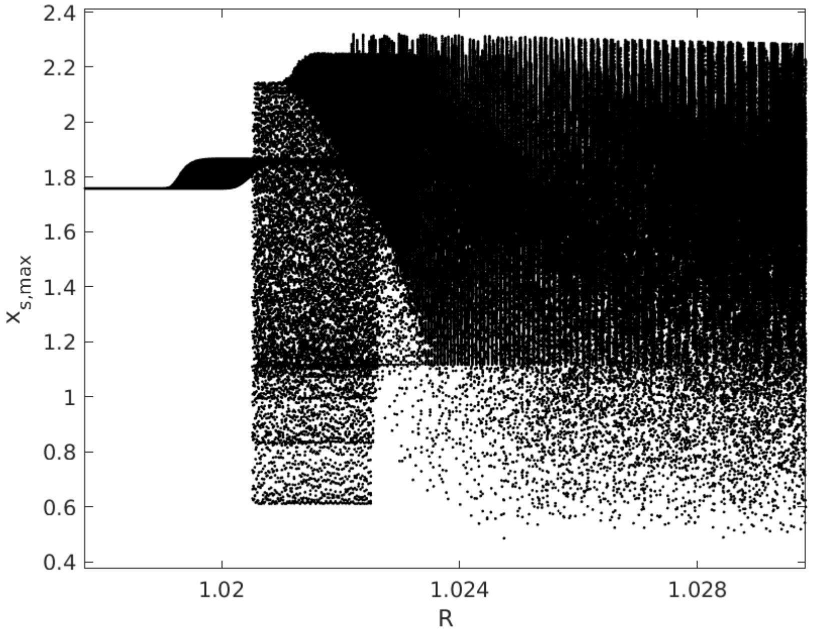

Figure 2. A plot showing the maximal values of x_s for the model system studied in the main paper as a function of the disease basic reproduction number R. The plot is analogous to fig. 1 (b). We see no signs of period doubling bifurcations, nor any other bifurcations, and we can therefore rule out that the transition to chaos takes place through this route. Instead, we believe that the transition is quasiperiodic. The trajectories near the transition, shown in fig. 3 (a) in the main paper, further support this conclusion. Parameter values used: $a = 7/400, b = 0.0208, c = 2, d = 0.3098$.

References

1. Eilersen, A. & Sneppen, K. The uneasy coexistence of predators and pathogens. *Under review* (2020).
